# Comparison of Female Genital Tract Cytokine and Microbiota Signatures Induced by Initiation of Intramuscular DMPA and NET-EN Hormonal Contraceptives - a Prospective Cohort Analysis

**DOI:** 10.3389/fimmu.2021.760504

**Published:** 2021-12-09

**Authors:** Smritee Dabee, Ramla F. Tanko, Bryan P. Brown, Rubina Bunjun, Christina Balle, Colin Feng, Iyaloo N. Konstantinus, Shameem Z. Jaumdally, Maricianah Onono, Gonasagrie Nair, Thesla Palanee-Phillips, Katherine Gill, Jared M. Baeten, Linda-Gail Bekker, Jo-Ann S. Passmore, Renee Heffron, Heather B. Jaspan, Anna-Ursula Happel

**Affiliations:** ^1^ Center for Global Infectious Disease, Seattle Children’s Research Institute, Seattle, WA, United States; ^2^ Institute of Infectious Disease and Molecular Medicine (IDM), University of Cape Town, Cape Town, South Africa; ^3^ Centre for the AIDS Programme of Research in South Africa (CAPRISA) Centre of Excellence in HIV Prevention, University of Cape Town, Cape Town, South Africa; ^4^ The Medical Research Centre, Institute of Medical Research and Medicinal Plant Studies (IMPM), Ministry of Scientific Research and Innovation, Yaoundé, Cameroon; ^5^ Namibia Institute of Pathology, Windhoek, Namibia; ^6^ Kenya Medical Research Institute, Kisumu, Kenya; ^7^ Desmond Tutu HIV Centre, Cape Town, South Africa; ^8^ Wits Reproductive Health and HIV Institute, University of the Witwatersrand, Johannesburg, South Africa; ^9^ Department of Global Health, University of Washington, Seattle, WA, United States; ^10^ Gilead Sciences, Foster City, CA, United States; ^11^ National Health Laboratory Service, Cape Town, South Africa

**Keywords:** female genital tract, inflammation, microbiome, hormonal contraception, HIV risk, Sub-Saharan Africa

## Abstract

**Background:**

Cervicovaginal inflammation, bacterial microbiota and hormonal contraceptives all influence sexual and reproductive health. To date, the effects of intramuscular depo-medroxyprogesterone acetate (DMPA-IM) versus injectable norethisterone enanthate (NET-EN) on vaginal microbiota or cytokines have not been compared back-to-back, although *in-vitro* data suggest that DMPA-IM and NET-EN have different pharmacokinetic and biologic activities. This study aimed at comparing the effects of DMPA-IM versus NET-EN initiation on cervicovaginal cytokines and microbiota in women at high risk for sexually transmitted infections (STIs) assigned to the respective contraceptives.

**Methods:**

We collected socio-demographic characteristics and vaginal samples from women initiating DMPA-IM (ECHO Trial; n = 53) and NET-EN (UChoose Trial; n = 44) at baseline and after two consecutive injections to assess cytokine concentrations by Luminex, vaginal microbiota by 16S rRNA gene sequencing, STIs, bacterial vaginosis (BV) and candidiasis.

**Results:**

Cytokine concentrations did not change significantly after initiating DMPA-IM or NET-EN, although NET-EN versus DMPA-IM-associated profiles were distinct. While the abundance of bacterial taxa associated with optimal and non-optimal microbiota fluctuated with DMPA-IM use, overall community composition did not significantly change with either contraceptive. HSV-2 serology, chlamydial infection, gonorrhoea and candidiasis did not influence the associations between contraceptive type and cervicovaginal cytokines or microbiota.

**Conclusions:**

Both DMPA-IM and NET-EN use did not lead to broad inflammatory or microbiota changes in the female genital tract of sub-Saharan African women. This suggests that NET-EN is likely a viable option for contraception in African women at high risk of BV and STIs.

## Introduction

Globally, women represent more than half of the 37 million people currently living with HIV (1). More than 600 000 new HIV infections occur each year among African women, with Sub-Saharan Africa (SSA) being the epicentre (1). The WHO has committed to ending the AIDS epidemic as a public health threat by 2030 (2), and to do so, strategies to mitigate HIV risk in women need to be identified.

The vulnerability of women to HIV infection is likely dependent on multiple factors (3). Robust evidence suggests that biological factors contribute to this risk, including sexually-transmitted infections (STIs) and bacterial vaginosis (BV), which may disrupt the mucosal barrier or induce host inflammatory pathways (4). Even in the absence of STIs, African women commonly appear to have higher levels of genital tract inflammation and higher abundances of non-optimal vaginal bacterial taxa (5–7), increasing their susceptibility to heterosexual HIV transmission (8). Prior to the Evidence for Contraceptive Options and HIV Outcomes (ECHO) Trial (9), observational studies suggested that the use of injectable progestogen contraceptives might increase HIV risk (10, 11). Such contraceptives, specifically intramuscular depo-medroxyprogesterone acetate (DMPA-IM) and intramuscular norethisterone enanthate (NET-EN), are the most commonly used contraceptives in SSA because they are convenient, long-acting, and reversible (12, 13).

While the ECHO Trial found that DMPA-IM did not significantly increase HIV risk compared to a copper intrauterine device (IUD) or a levonorgestrel (LNG) implant in SSA women (9), it did not compare risk associated with NET-EN use. There are various biological mechanisms by which contraceptive use could increase HIV risk, including changes in immune cell activity (14), inflammatory and chemotactic cytokine levels, or the vaginal microbiota (8). Previous studies have explored the effects of DMPA-IM and NET-EN initiation on cervicovaginal microbiota and cytokines, but comparisons cannot be done given the differences in methodology used for these assessments. As such, the UChoose Trial included women randomized to NET-EN but compared cervicovaginal cytokines and microbiota to women randomized to the Nuvaring^®^ and combined oral contraceptives (15). Further, the data for the ECHO and UChoose trials were generated using different Luminex panels and different 16S rRNA gene sequencing methods, thus limiting the conclusions that can be made from these results regarding the differentials effects of DMPA-IM or NET-EN on cervicovaginal cytokines and bacterial microbiota. Another caveat is that neither trial directly compared the effects of NET-EN and DMPA-IM use, which have different pharmacokinetic, biological and epidemiologic characteristics (16), on genital inflammation and bacterial microbiota. Limited clinical data have suggested changes in immunologic activity following DMPA-IM and NET-EN initiation (17–19), but inter-study variation and differences in study design and analyses approaches diminish the ability to determine the degree to which DMPA-IM and NET-EN act differently.

Therefore, we compared cytokine and microbial signatures in the female genital tract (FGT) of SSA women initiating DMPA-IM (ECHO Trial) versus women initiating NET-EN (UChoose Trial), using identical Luminex and 16S rRNA gene sequencing methods, while accounting for differences in demographic, behavioral and biological factors of the women enrolled through separate trials. Defining differences in cytokines and vaginal bacterial microbiota composition in the FGT in response to DMPA-IM versus NET-EN initiation will provide important insights into the relative safety of NET-EN in SSA women who are at high risk of STI and HIV acquisition.

## Materials and Methods

### Study Design

Samples included in this study were collected as part of the ECHO (clinicaltrials.gov/NCT02550067) and UChoose (clinicaltrials.gov/NCT02404038) Trials. Both trials were implemented in accordance with Good Clinical Practice. The ECHO Trial was approved by the Research Ethics Committees at the University of Cape Town (HREC 371/2015), University of Witwatersrand (HREC PRC 141112), KEMRI (SERU/CMR/P0014/3109), University of Washington (STUDY00000261), and FHI360 (523201). The UChoose Trial was approved by the Research Ethics Committee at the University of Cape Town (HREC 801/2014). All participants 18 years or older provided informed consent, while informed assent from the participant and informed consent from a parent or legal guardian were obtained for participants younger than 18 years old.

The ECHO Trial was a randomized, multicentre, open-label trial carried out at 12 research sites in SSA to evaluate HIV incidence among non-pregnant, HIV-seronegative women aged 16–35 years who self-reported not using injectable, intrauterine, or implantable contraception for the previous 6 months. Women were randomized to copper-IUD, the LNG-implant or DMPA-IM (150 mg of depot medroxyprogesterone acetate), provided on site at enrolment and then every three months for 18 months. Inclusion criteria for the parent trial were previously described (9). At three sites, the Emavundleni Clinical Research Site, Cape Town, South Africa, the Wits Reproductive Health and HIV Institute (WRHI), Johannesburg, South Africa and the Kenya Medical Research Institute (KEMRI), Kisumu, Kenya, participants were sequentially recruited into a nested sub-study of mucosal immunology. From this subset of participants, women assigned to DMPA-IM were included in the current analysis. Samples at baseline and after two injections of DMPA-IM (months zero and six) were included in this analysis.

The UChoose Trial was an open-label, randomized crossover trial at the Masiphumelele Clinical Research Site, Cape Town, South Africa, aiming to evaluate the feasibility of hormonal contraceptive options as proxy for HIV prevention methods among non-pregnant, HIV-seronegative adolescents aged 15-19 years who were either contraception-naïve, or on a short-term contraceptive method and willing to change to another method. Detailed eligibility criteria have been previously described (20). Briefly, adolescents were randomized to a combined contraceptive intravaginal ring, combined oral contraceptive pills or the bi-monthly injectable hormonal contraception NET-EN (200mg of norethisterone enantate). From these participants, only women assigned to NET-EN were included in the current analysis. Samples at baseline and after two injections of NET-EN (months zero and four) were included in this analysis.

### Study Procedures

In both trials, detailed interviewer-assisted questionnaires assessing demographics, medical and obstetric history, and sexual behaviour were completed. At baseline and after two contraceptive injections, cervicovaginal secretions were collected using menstrual cups inserted for at least 30 minutes. Clinicians removed the menstrual cup prior to speculum insertion and immediately placed the cup into a sterile tube. Menstrual cup secretions were extracted by centrifuging at 453g at 4°C for 10min upon reaching the laboratory, and diluted 1:4 in sterile phosphate buffered saline (PBS). Using a speculum, vaginal swabs were collected from the lateral vaginal wall for microbiota analysis. The swab was placed into a sterile vial with Digene^®^ transport media for DNA extraction. All samples were immediately placed at 4°C, transported to the laboratory within 4hrs of collection, and stored at -80°C until analysis. Other samples collected include genital swabs for STI and BV testing, and blood for herpes simplex virus 2 (HSV-2) serology.

### STI and BV Diagnosis

In both trials, blood was collected at baseline and serology was performed to screen for IgG antibodies to herpes simplex virus 2 (HSV-2) gG2 using an enzyme-linked immunosorbent assay (HerpeSelect, Focus Diagnostics, USA). Genital swabs were collected to test for STIs (*Chlamydia trachomatis* and *Neisseria gonorrhoeae*) at baseline (pre-contraceptive initiation), and for Nugent-BV and candidiasis at baseline and after two injections of either NET-EN or DMPA-IM. In the UChoose Trial, vulvovaginal swabs were tested for *C. trachomatis* and *N. gonorrhoeae* by multiplex PCR, as previously described (21). In the ECHO Trial, the GeneXpert Instrument Systems platform (Cepheid Inc., US) with the Abbott Real Time PCR assay (Abbott Molecular, US) was used to test for *C. trachomatis* and *N. gonorrhoeae* in endocervical swabs at the South African sites, while the Panther System (Hologic Inc., US) was used in Kenya. For both studies, microscopic slides with vaginal smears were Gram-stained for Nugent scoring (to diagnose Nugent-BV, where participants with a Nugent score 0-3 were BV-negative, those with a Nugent score of 7-10 were BV-positive and those with an intermediate microbiota had a Nugent score of 4-6) and candidiasis screening (*Candida* hyphae and spores). Participants were provided treatment for curable STIs, using both syndromic and aetiological diagnoses. BV was treated syndromically in accordance with the local syndromic management guidelines.

### Measurement of Cervicovaginal Cytokines

For this analysis, specimens from the UChoose and ECHO Trials included here were assayed as part of the same Luminex runs, using identical assay lot numbers. After filtering (0.2μM cellulose acetate filters) cervicovaginal fluid, the concentrations of IL-1β, IL-6, TNF-α and IL12(p70) (inflammatory cytokines), CXCL8/IL-8, Exotaxin, CXCL10/IP-10, CCL2/MCP-1, CCL3/MIP-1α, CCL4/MIP-1β and CCL5/RANTES (chemokines), IL-2, IL-4, IL-5, IL-13, IL-15, IL-17 and IFN-γ (adaptive cytokines), IL-7, IL-9, FGF-basic, G-CSF, GM-CSF, PDFG-BB, and VEGF (growth factors), and IL-1RA and IL-10 (regulatory cytokines) were measured by Luminex using the Bio-Plex™ Pro Human cytokine 27-plex assay (Bio-Rad Laboratories Inc^®^, USA) according to the manufacturer’s instructions, alongside intra- and inter-plate controls, and acquired using a Bio-Plex Suspension Array Reader (Bio-Rad Laboratories Inc^®^, USA). The Bio-plex manager software (version 4) was used for data analysis and a 5 Parameter Logistic (5 PL) regression formula was used to calculate cytokine concentrations from the standard curves. Cytokine concentrations below the lower limit of detection of the assay were reported as the mid-point between zero and the lowest concentration measured for each cytokine.

### 16S rRNA Gene Sequencing and Analysis

For this analysis, specimens from the UChoose and ECHO Trials included here were assayed as part of the same library preparation and sequencing runs. Lateral vaginal wall swabs (Digene^®^) were slowly thawed on ice, shaken at 100rpm for 3 minutes, and 250μl of suspension was used for DNA extraction. Bacterial gDNA extraction was carried out using the Qiagen DNeasy Powersoil HTP 96 kit according to the manufacturer’s instructions. If there was insufficient volume in the sample vial, 550μl of sterile PBS was added to the vial, which was then shaken for another 3 minutes at 100rpm, and 250μl of suspension transferred for DNA extraction. After DNA extraction, sequencing of the V3-V4 hypervariable region of the bacterial 16S rRNA gene was carried out as described previously (22) except using 357F/806R primers (**Supplementary Table 1**). Samples were amplified in triplicates. Amplicons were purified using AMPure XP beads (Beckman Coulter) and pooled in equal mass amounts to be sequenced using the Illumina MiSeq platform (300bp paired-end, V3). Following demultiplexing, raw reads were processed and classified using DADA2 (23). Samples with <2000 reads were excluded from further analyses. Taxonomic annotation was carried out using the Ribosomal Database Project’s (RDP) Naïve Bayesian classifier (24). Sequence classification was trained against an updated version of the Silva training set version 132 (25), available at https://github.com/itsmisterbrown/updated_16S_dbs. Run-specific contamination filtering was performed using microfiltR (https://github.com/itsmisterbrown/microfiltR). Sequencing runs were merged using custom scripts (https://github.com/itsmisterbrown/marker_gene_dataset_merging_functions).

### *Post-hoc* Power Calculations

With the sample size (n=44 NET-EN, n=53 DMPA-IM) of this study, the minimal difference detectable with 80% power at α=0.05 would have been a 2.45-fold increase in the proportion of women with genital inflammation (when binarizing genital inflammation as absent/present based on the upregulation of ≥5/8 cytokines to above their 75^th^ percentile) (8, 26) assigned to DMPA-IM versus NET-EN.

### Statistical Analyses

All statistical analyses were performed in RStudio Version 3.6 and STATA version 11.0 (StataCorp, Texas, US). Cross-sectional differences in baseline group characteristics were tested using Pearson´s Chi-squared test or Fisher’s exact test (when the expected value was <5) for count data. The unpaired Mann-Whitney U-test was applied for differences in medians. Multivariate logistic regression analyses were performed using the cytokine and microbiota data, and confounders were identified using a step-up model-building strategy (including site, age, sexual risk behaviour, bacterial STIs, HSV-2 serology, Nugent-BV and candidiasis). Adjusted R^2^ and Akaike information criterion (AIC) values were compared as a measure of model fit. For our population, the most robust model was adjusted for baseline HSV-2 serology, *C. trachomatis, N. gonorrhoeae* and candidiasis. A false discovery rate step down procedure (27) was used to adjust for multiple comparisons. 95% confidence intervals and p values of ≤0.05 were used to assess statistical significance. The presence of genital inflammation in women was defined by their relative concentrations of eight proinflammatory cytokines and chemokines (IL-1β, IL-6, CXCL8/IL-8, CXCL10/IP-10, CCL2/MCP-1, CCL3/MIP-1α, CCL4/MIP-1β and TNF-α), with genital inflammation present if five or more of these eight markers were above the 75th percentile, as previously described (8, 26). Vaginal microbial community state types (CSTs) were generated using partitioning around medoids (PAM) clustering using the cluster package (28). Downstream microbiota data analysis, including ecological diversity metrics, ordination and fold change differences, was performed in R (v3.6.0) using the packages phyloseq (29), NMF (30), metagenomeSeq (31), vegan (32), and DESeq2 (33). The permutational multivariate analysis of variance (PERMANOVA) test was used to assess the heterogeneity of dispersion between groups of samples. The Data Integration Analysis for Biomarker discovery using Latent cOmponents (DIABLO) framework, as part of the mixOmics R Bioconductor package (34), was used for multi-omics analyses integrating the microbial and inflammation data. Cytokines and bacterial taxa that accounted for the highest degree of variance between the two study arms were selected via least absolute shrinkage and selection operator (LASSO) followed by sparse partial least-squares discriminant analysis (PLS-DA).

## Results

### Characteristics of Study Participants

Among the 97 HIV-seronegative SSA women included in this analysis, 44 were initiating NET-EN (Cape Town, South Africa) and 53 were initiating DMPA-IM (Cape Town [n=23] and Johannesburg [n=15], South Africa and Kisumu, Kenya [n=15]). In accordance with the design of the parent trials described earlier, women initiating DMPA-IM were older than women initiating NET-EN (median 24 [interquartile range (IQR) 22-29] vs. 17 [IQR 16-18] years, respectively; p<0.0001). Likely due to these age differences, more women initiating DMPA-IM had previously been pregnant (81.1% vs. 11.4%, p<0.0001), had less alcoholic drinks per week (median 0 [IQR 0-3] vs. 3 [IQR 0-5], p=0.011) and a higher HSV-2 seroprevalence (64.2% vs. 25.0%, p<0.0001), indicating previous infection with HSV-2. Possibly also age-related, prevalence of active *C. trachomatis* and *N. gonorrhoeae* infections tended to be higher in women initiating NET-EN (14/44; 31.8%) compared those initiating DMPA-IM (9/53; 17.0%; p=0.141), as did Nugent-BV prevalence (40.9% vs. 25.0%; p=0.085), while sexual risk behaviour was comparable between groups (**Table 1**). All but one participant per group continued to use their assigned intervention through follow-up.

**Table 1:** Participant demographics, female genital tract health and sexual risk behaviour.

	DMPA-IM	NET-EN	p
n	53	44	
Demographics			
Site [n(%)]			**<0.001**
Masiphumelele Clinical Research Site, Cape Town, South Africa	0 (0.0)	44 (100.0)^#^	
Emavundleni Clinical Research Site, Cape Town, South Africa	23 (43.4)*	0 (0.0)	
KEMRI, Kisumu, Kenya	15 (28.3)^$^	0 (0.0)	
WRHI, Johannesburg, South Africa	15 (28.3)^$^	0 (0.0)	
Age [(median [IQR)]	24 (21- 28)	17 (16 – 18)	**<0.001**
BMI [median (IQR)]	24.4 (22.0 - 29.1)	25.4 (23.4 - 29.4)	0.494
Female genital tract health [n(%)]			
Baseline HSV-2 seroprevalence^a^	34 (64.2)	11 (25.0)	**<0.001**
Baseline *N. gonorrhoea^b^*	4 (7.5)	5 (11.4)	0.769
Baseline *C. trachomatis^b^*	8 (15.1)	11 (25.0)	0.334
Baseline Any bacterial STI	9 (17.0)	14 (31.8)	0.141
Baseline Nugent-BV			0.198
Negative	34 (65.4)	21 (47.7)	
Intermediate	5 (9.6)	5 (11.4)	
Positive	13 (25.0)	18 (40.9)	
Baseline Candidiasis	1 (1.9)	3 (6.8)	0.494
Visit 2 Nugent-BV			0.708
Negative	28 (60.9)	23 (52.3)	
Intermediate	1 (2.2)	1 (2.3)	
Positive	17 (37.0)	20 (45.5)	
Visit 2 Candidiasis	3 (6.7)	7 (15.9)	0.296
Visit 2 Sexual Risk Behaviour			
Visit 2 Disclosed study to partner [n(%)]	44 (83.0)	33 (75.0)	0.472
Visit 2 Know HIV status of primary partner [n(%)]	38 (71.1)	31 (73.8)	1.000
Visit 2 Participant has multiple partners [n(%)]	5 (9.4)	4 (9.1)	1.000
Visit 2 Participant’s partner has multiple partners [n(%)]			0.336
Yes	8 (15.1)	7 (15.9)	
Don’t know	28 (52.8)	17 (38.6)	
Visit 2 Condom use during last sex act [n(%)]	26 (49.1)	27 (62.8)	0.225
Visit 2 Number vaginal sex acts per week [mean (SD)]	1.73 (2.15)	1.86 (1.23)	0.763
Visit 2 Transactional sex [n(%)]	4 (7.5)	0 (0.0)	0.109
Visit 2 Number drinks per week [median [IQR)]	0 (0- 3)	3 (0- 5)	**0.011**
Visit 2 Previously pregnant [n(%)]	43 (81.1)	5 (11.4)	**<0.001**

^#^No Luminex data available for n=1, no 16S rRNA gene sequencing data available of n=5.

*No Luminex data available for n=6, no 16S rRNA gene sequencing data available of n=3.

^$^No 16S rRNA gene sequencing data available of n=1.

BMI, body mass index; HSV, herpes simplex virus; IQR, interquartile range; SD, standard deviation; STI, sexually transmitted infections; KEMRI, Kenya Medical Research Institute; WRHI, Wits Reproductive Health and HIV Institute .

a Seroprevalence was assessed in blood by ELISA.

b Active infection was assessed in genital swabs by PCR assays.

Bold values indicate statistical significance after adjustment for multiple comparisons.

### DMPA-IM or NET-EN Use Did Not Lead to Increased Genital Inflammation

**Figure 1:**
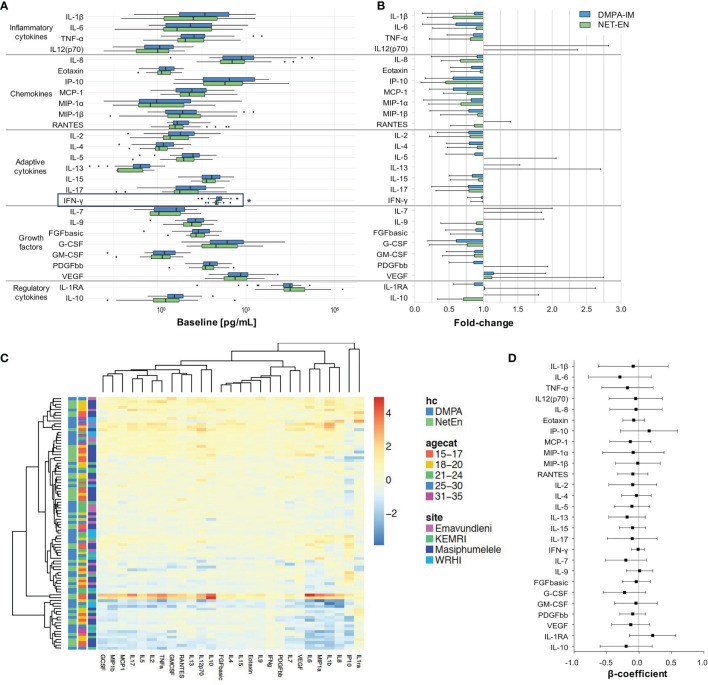
Genital cytokine changes in women assigned to DMPA-IM and NET-EN. **(A)** Comparison of baseline log_10_ cytokine concentrations between NET-EN and DMPA-IM users. p ≤ 0.05 were considered statistically significant and are represented by an *. **(B)** Fold-change in log_10_ cytokine concentrations from baseline to Visit 2. Participants using DMPA-IM are shown in blue and those using NET-EN are shown in green. **(C)** Heatmap of the change in log_10_ cytokine concentrations from baseline to Visit 2 using unsupervised clustering. The color key indicates the change in cytokines concentrations (yellow=no change, orange/red=increase, blue=decrease). Annotations include the participant’s assigned contraceptive type, age category, and enrollment site. **(D)** Multivariate linear regressions showing the association between change of absolute genital cytokine concentrations from baseline to Visit 2 in women using DMPA-IM as compared to NET-EN. Each association is shown as a β-coefficient and the error bars are the 95% CI.

At baseline, cervicovaginal cytokine concentrations were largely similar between women initiating DMPA-IM or NET-EN. Possibly due to differences in lived experiences of participants from different sites, some cytokines differed by recruitment site, age group, STI or Nugent-BV status, using univariate analyses prior to adjustment for multiple comparisons (**Supplementary Table 2**). However, IFN-γ was the only cytokine that remained significantly higher in women initiating DMPA-IM (120.14 [IQR 98.95 - 149.03] pg/ml) compared to those initiating NET-EN (98.78 [IQR 89.52 - 112.95] pg/ml; adjusted p=0.039) after adjusting for multiple comparisons (**Figure 1A**). We thus proceeded to only conduct paired longitudinal analyses that assessed the change in cervicovaginal cytokine concentrations from baseline to follow-up after two injections of the respective contraceptive.

The fold-changes in individual cervicovaginal cytokine concentrations from baseline to after two consecutive contraceptive injections were largely similar between women using DMPA-IM and those using NET-EN (**Figure 1B**). Further, the absolute differences in cytokine concentrations from baseline to follow-up did not differ between women using DMPA-IM and those using NET-EN (**Supplementary Figure 1B**). Albeit not significantly, cervicovaginal fluid from women using DMPA-IM had decreased levels of 20/27 cytokines compared to matched baseline samples, with CXCL10/IP-10 (1.8-fold), CCL2/MCP-1 (1.8-fold), IL-6 (1.6-fold) and GCSF (1.6-fold) being the most notable examples (**Figure 1B**). NET-EN use was also associated with a decrease in 21/27cytokines, including CXCL10/IP-10 (2.2-fold), IL-1β (1.8-fold), CXCL8/IL-8 (1.5-fold) and CCL3/MIP-1α (1.5-fold). Both contraceptives increased VEGF 1.1-fold (**Figure 1B**).

No clustering by contraceptive type, age category or site was observed when assessing change in log_10_ cytokine concentrations from baseline to follow-up using unsupervised clustering (**Figure 1C**). When binarizing genital inflammation as absent/present (based on the upregulation of ≥5/8 cytokines to above their 75^th^ percentile) (8, 26), the proportion of women with genital inflammation decreased in both groups (by 8.5% in DMPA-IM and by 2.2% in NET-EN users) from baseline to follow-up, albeit non-significantly. Overall, these data indicate that neither DMPA-IM nor NET-EN use changed cervicovaginal cytokine profiles notably.

The influence of possible confounders, such as site, age, marital status, number of partners, number of sexual acts per week, and condom use on these findings were assessed in multivariate linear regressions. We found that these variables did not influence the observed associations between contraceptive type and change in cytokine concentrations. After adjusting for baseline HSV-2 serology, active bacterial STIs and candidiasis, DMPA-IM use resulted in a general modest decrease in absolute cytokine concentrations from baseline to follow-up compared to NET-EN use (reference category), albeit not significantly (**Figure 1D**).

### Age or Geography Did Not Significantly Influence the Impact of DMPA-IM or NET-EN Use on Cervicovaginal Cytokines

Due to trial design differences, the age of women initiating DMPA-IM and NET-EN differed, and since participant age may influence responses to hormonal contraceptive initiation, we conducted a sensitivity analysis that included only participants ≤24 years old (DMPA-IM: n=27; NET-EN: n=43). In agreement with observations from the complete dataset, participants generally experienced a non-significant decrease in absolute cytokine concentrations from baseline to after two contraceptive injections and this did not differ between DMPA-IM and NET-EN users (**Supplementary Figure 2A**). Fold-decreases from baseline to follow-up were of similar magnitude in the same cytokines as in the full dataset (**Supplementary Figure 2B**). As in the complete data set, change in absolute cytokine concentrations from baseline to after two contraceptive of women aged ≤24 years using DMPA-IM differed slightly to that of women using NET-EN (reference) after adjusting for confounding factors, albeit not significantly (**Supplementary Figure 2C**).

To account for differences in geography, a sensitivity analysis including women from Cape Town, South Africa only (DMPA-IM: n=17; NET-EN: n=43) was carried out. Similar to findings from all sites, cytokine concentrations were not significantly elevated after two contraceptive injections in DMPA-IM or NET-EN users compared to baseline, and changes in absolute cytokine concentrations from baseline to follow-up did not differ significantly by contraceptive type (**Supplementary Figure 3A**). Similar to when including all sites, a fold-decrease in cytokine concentrations from baseline to follow-up was observed for most cytokines in DMPA-IM and NET-EN users (**Supplementary Figure 3B**), although DMPA-IM use was associated with a non-significant increase in CXCL8/IL-8 (1.4-fold), VEGF (1.4-fold), PDGFbb (1.3-fold), and IL12p70, CCL5/RANTES, IL-2, FGFbasic, GM-CSF and IL1RA (all 1.1-fold), while NET-EN initiation was associated with a 1.1-fold increase in VEGF only. However, after adjusting for confounding factors, changes in absolute cytokine concentrations from baseline to follow-up were comparable among DMPA-IM and NET-EN users (**Supplementary Figure 3C**) when restricting this analysis to isiXhosa women from Cape Town, South Africa.

Together, these data suggest that NET-EN and DMPA-IM use do not have an adverse impact on cytokine profiles in the FGT, also when accounting for differences in participants’ age and geography.

### DMPA-IM or NET-EN Use Did Not Cause Significant Changes in Clinical Microbial Diagnoses

Women were assessed for both Nugent-BV and candidiasis at baseline and after two injections of their respective contraceptive. While baseline Nugent-BV rates tended to be lower among women who were subsequently assigned to DMPA-IM (25.0%) compared to those who were subsequently assigned to NET-EN (40.9%, p=0.085), Nugent-BV prevalence was more comparable between NET-EN (45.5%) and DMPA-IM (37.0%, p=0.177) users after two consecutive injections of the respective contraceptive. Candidiasis was generally less prevalent but increased 2-3-fold in both groups compared to baseline (1.9% at baseline vs. 6.7% at follow-up for DMPA-IM users, p=0.308; and 6.8% at baseline vs. 15.9% at follow-up for NET-EN users, p=0.179).

### Neither DMPA-IM nor NET-EN Use Caused Broad Shifts in the Vaginal Microbiota Composition

At baseline, participant age did not influence the genital microbiota distribution (Bray-Curtis distances); however, the overall microbial community composition differed significantly between the NET-EN and DMPA-IM arms (**Supplementary Figure 4A**; PERMANOVA p=0.004) and between sites within the DMPA-IM arm (**Supplementary Figure 4C**; PERMANOVA p=0.040). Similarly, within-participant α-diversity (assessed using Shannon indices) in the DMPA-IM arm differed significantly to the NET-EN arm (**Supplementary Figure 4B**; p=0.001). Due to these baseline differences in genital microbiota composition, only paired longitudinal changes were analysed further (DMPA-IM: n=48; NET-EN: n=39).

**Figure 2:**
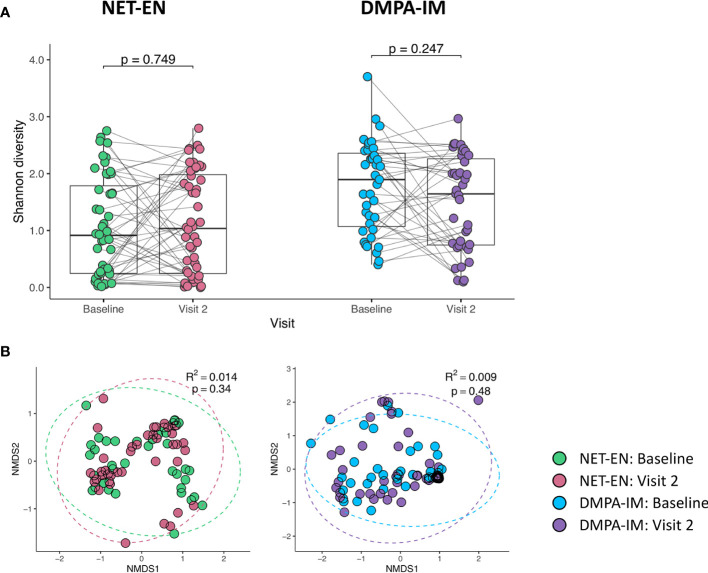
Transitions in overall microbial composition after two injections in women using NET-EN and DMPA-IM. **(A)** Within participant (⍺-diversity) using Shannon diversity metric and **(B)** between participant bacterial diversity (β-diversity) using principal component analysis of Bray-Curtis differences at baseline and Visit 2. NMDS, Non-metric Multidimensional Scaling.

Based on these paired pre (baseline)-post (follow-up) analyses, we found no longitudinal change in α-diversity (within-participant Shannon diversity; **Figure 2A**) or β-diversity (between-participant Bray-Curtis distances, **Figure 2B**) from baseline to after two injections of either contraceptive, suggesting that there were no notable shifts in the vaginal microbiota composition with neither DMPA-IM nor NET-EN use. To confirm that participant age, and particularly site, did not influence our findings, we carried out sensitivity analyses only including participants aged ≤ 24 years or only including participants from Cape Town. We found no difference in α- (**Supplementary Figure 5A**) or β-diversity (**Supplementary Figure 5B**) in paired analyses from baseline to follow-up with the initiation of DMPA-IM or NET-EN among women aged ≤ 24 years (DMPA-IM: n=30; NET-EN: n=39) or among those from Cape Town only (DMPA-IM: n=20; NET-EN: n=39; **Supplementary Figures 5C, D**) (all p>0.05).

**Figure 3:**
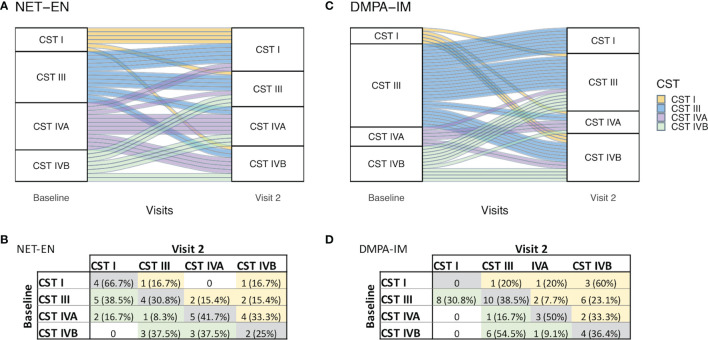
Shift in community state type (CST) from baseline to after two NET-EN **(A)** and DMPA-IM **(C)** injections. CST I: *L. crispatus*-dominant; CST III: *L. iners*-dominant; CST IVA: Diverse, BV-associated bacteria; CST IVB: Diverse, higher abundance of *L. iners* and *G*. *vaginalis*. Each line represents the transition for one participant **(A, C)**. Frequency tables of the number of participants assigned to each CST at baseline vs. Visit 2 for NET-EN **(B)** and DMPA-IM **(D)** users.

To assess whether DMPA-IM use influenced community composition differently to NET-EN use, PAM clustering of Bray-Curtis distances was carried out to categorize women into one of four community state types (CSTs), based on bacterial species proportions (**Supplementary Figure 4D**). CST-I and -III were *Lactobacillus*-dominant CSTs, with CST-I being dominated by *L. crispatus* and CST-III by *L. iners*. CST-IVA had a higher diversity of BV-associated bacteria and CST-IVB had a higher abundance of *L. iners* and *Gardnerella vaginalis*. CST-I was associated with the least, and CST-IVB with the highest overall α-diversity (**Supplementary Figure 4E**). At baseline, there were no differences in the distribution of CSTs between women in the NET-EN or DMPA-IM groups (p=0.106; **Figure 3**). Overall, 35.4% of DMPA-IM and 38.5% of NET-EN users remained in the same CST category as at baseline after two injections (**Figures 3A, C**). About 15% of women moved from a more diverse to a lactobacilli-dominant state in both arms, while 25% (12/48) women moved from a lactobacilli-dominant CST to a more diverse CST in the DMPA-IM arm compared to 13% (5/39) in the NET-EN arm (p=0.154) (**Figures 3B, D**).

### DMPA-IM and NET-EN Use Caused Subtle Shifts in Bacterial Abundances

**Figure 4:**
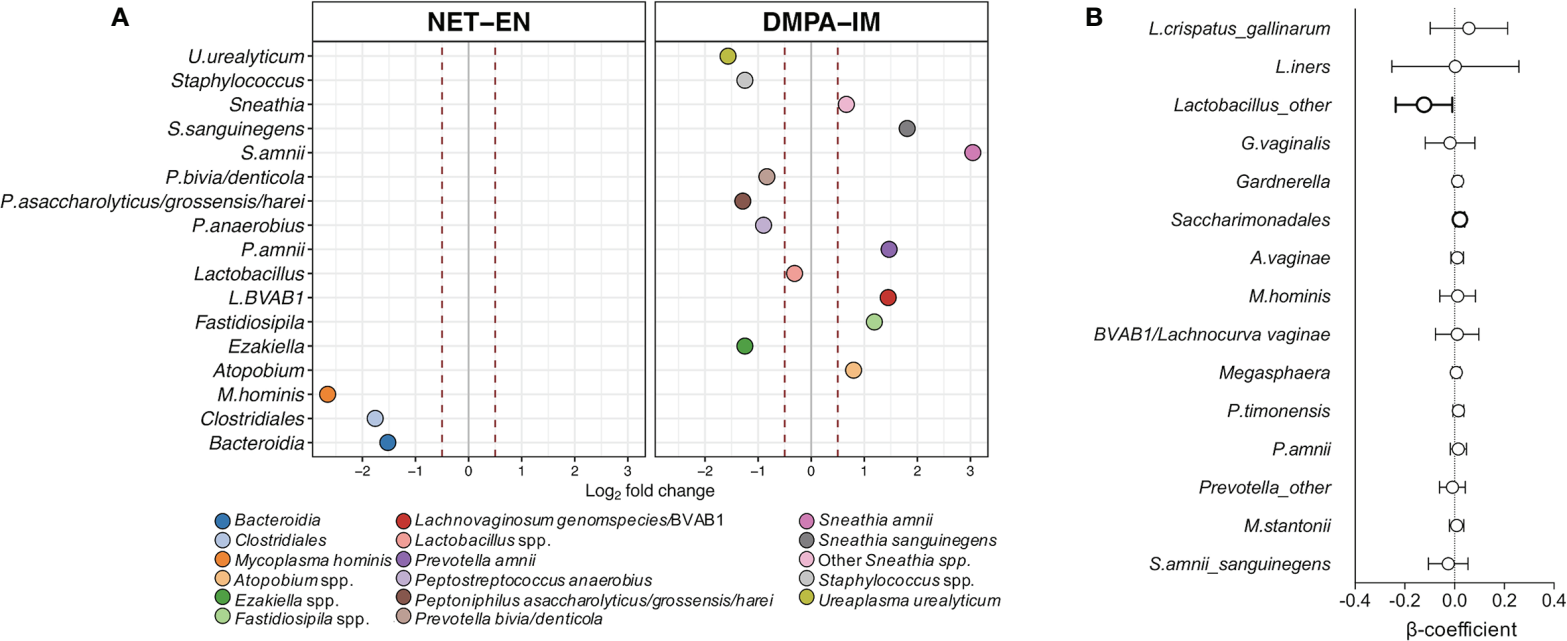
Change in abundance of individual bacterial taxa with DMPA-IM and NET-EN use. **(A)** Fold change differences in the abundance of specific bacterial taxa in matched samples from baseline to after two rounds of injections with either NET-EN or DMPA-IM. Dots on the right- and left-hand side of the grey line represent a fold change decrease or increase, respectively. The red dotted lines represent a 0.5 fold change difference in abundance. Only differential abundant taxa with a p ≤ 0.01 after adjusting for multiple comparisons were included. **(B)** Multivariate linear regressions showing the association between the most prevalent bacterial taxa in women using DMPA-IM as compared to NET-EN users. Each association is shown as a β-coefficient and the error bars are the 95% confidence interval (CI). The associations shown in bold were statistically significant before correcting for multiple comparisons. p values ≤0.05 were considered statistically significant.

To assess whether specific bacterial taxa differed by contraceptive type, fold change differences in abundance of individual bacterial taxa were determined for both groups, adjusting for geographic site. DMPA-IM use was associated with a markedly larger number of bacterial shifts compared to NET-EN use (**Figure 4A**; **Supplementary Table 3**; all p ≤ 0.01) from baseline to follow-up. With NET-EN use, there was a statistically significant decrease in *Mycoplasma hominis* (log_2_ 2.66-fold), *Clostridiales* (log_2_ 1.76-fold), and *Bacteroidia* spp. (log_2_ 1.52-fold) abundances at follow-up compared to matched baseline samples. In contrast, DMPA-IM use was accompanied by an increase in several BV-associated bacteria, including *Sneathia* spp. (log_2_ 0.66-fold), *Atopobium* spp. (log_2_ 0.80-fold), *Fastidiosipila* spp. (log_2_1.19-fold), BVAB1/*Lachnocurva vaginae* (log_2_1.45-fold), *Prevotella amnii* (log_2_ 1.47-fold), *Sneathia sanguinegens* (log_2_ 1.81-fold) and *Sneathia amnii* (log_2_ 3.04-fold). On the other hand, there was a decrease in bacteria associated with both BV and genital health: *Ureaplasma urealyticum* (log_2_ 1.57-fold), *Peptoniphilus asaccharolyticus/grosssensis/harei* (log_2_ 1.29-fold), *Staphylococcus* spp. (log_2_ 1.25-fold), *Ezakiella* spp. (log_2_ 1.25-fold), *Peptostreptococcus anaerobius* (log_2_ 0.90-fold), *Prevotella bivia/denticola* (log_2_ 0.84-fold) and *Lactobacillus* spp. (log_2_ 0.31-fold) abundances.

To confirm that these associations between contraceptive type and genital microbiota were not confounded by other variables, multivariate linear regressions comparing standardised changes in abundances of the most prevalent bacterial taxa in DMPA-IM users compared to NET-EN users (with the latter being considered the reference category), adjusting for active bacterial STIs, candidiasis, and HSV-2 seroprevalence were conducted. We found that DMPA-IM use was associated with a decrease in *Lactobacillus* spp. (non-*L. iners/L. crispatus*; β=-0.12; p=0.035) and an increase in *Saccharimonadales* spp. (β=0.02; p=0.02) relative to NET-EN use, although not after adjusting for multiple comparisons (**Figure 4B**; adjusted p=0.26 and p=0.30, respectively).

Together, these data suggest that NET-EN and DMPA-IM use do not have significant non-optimal effects on the vaginal microbiota, even when accounting for differences in participants’ age and geography.

### No Overall Differential Effect in the Genital Milieu With Either DMPA-IM or NET-EN Use

**Figure 5:**
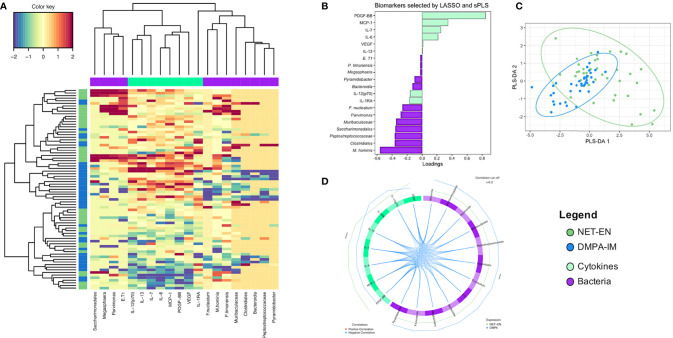
The overall integrated inflammation and microbial profile of women using NET-EN and DMPA-IM. NET-EN and DMPA-IM users are represented on the left in blue and dark green, respectively. **(A)** Unsupervised clustering with cytokine and bacterial biomarker clusters shown on the top of the heatmap in lime green and purple, respectively. The colour key shows the range of correlation values. **(B)** Loadings plot showing the contribution of the different biomarkers selected by LASSO, followed by sparse PLS-DA, that explain the highest variance in the comparison of NET-EN vs DMPA-IM. **(C)** Sparse PLS-DA with L_1_ penalizations showing the overlap between the NET-EN and DMPA-IM groups. **(D)** Circos plot showing the correlations among the selected biomarkers. Blue lines represent a negative correlation. Only associations above the threshold correlation cutoff of 0.3 were included. The outer green and blue lines represent NET-EN and DMPA-IM use, respectively.

Finally, we integrated cervicovaginal cytokine and microbiota data to identify genital biomarkers that were distinct between DMPA-IM and NET-EN users (**Figure 5**). LASSO and sparse-PLS-DA were used to identify and classify specific cytokines and bacterial taxa that explained the most variance between NET-EN and DMPA-IM use. The identified biomarkers included eight cytokines (VEGF, IL-12(p70), IL-1RA, IL-13, IL-7, IL-6, CCL2/MCP-1 and PDGF-BB) and 12 bacterial taxa (*M. hominis, Saccharimonadales, Parvimonas, Muribaculaceae, Peptostreptococcaceae, Clostridiales, Fusobacterium nucleatum, Eggerthella T1, Bacteroidia, Megaspheara, Prevotella timonensis*, and *Pyramidobacter*) (**Figures 5B, D**). DMPA-IM use appeared to mostly be influenced by changes in the microbiota while cytokine changes were more influential among NET-EN users, although none were significant (**Figures 5B, D**), and there was no distinct separation between the two groups using sPLS-DA (**Figure 5C**). Based on these biomarkers, we found no clustering between DMPA-IM and NET-EN users, indicating that neither contraceptive had a marked influence on vaginal cytokines or bacteria (**Figure 5A**).

## Discussion

Providing women of reproductive age with safe contraceptive options is crucial for women’s health. In SSA, where there is a strong overlap between progestogen-only injectable use and STI and HIV risk, it is important to understand if, and how, these contraceptives could impact the risk of adverse sexual health outcomes. Based on the ECHO Trial results (9), the WHO concluded that women, including those at high risk of HIV acquisition, could safely use any form of reversible contraception evaluated in the Trial, including progestogen-only injectables, although the trial did not evaluate the effect of the progestogen-only injectable NET-EN on HIV risk. As DMPA-IM and NET-EN contain two different progestins and may have differing biological effects (16), we compared changes in cervicovaginal inflammatory markers and bacteria in women assigned to DMPA-IM versus those assigned to NET-EN, as both genital inflammation (8) and microbiota (35) have been linked to HIV risk.

Balle et al. found, in a sub-study of the UChoose Trial, that NET-EN initiation did not significantly increase genital cytokine concentrations that have previously been associated with HIV risk (15). In this analysis, we demonstrate that the changes induced by DMPA-IM did not differ significantly from those induced by NET-EN use. Rather than increasing genital cytokines, initiation of both progestogen-only injectables led to a decrease of most cytokines evaluated. These findings are in agreement with some previous studies (17, 19, 36) that found a decrease or no change in cervicovaginal proinflammatory cytokines in women using progestogen-only injectable contraceptives, although other studies have found associations between DMPA-IM use and increases in genital cytokines (18), or systemic levels of IFN-γ, IL-10, CXCL-9 and sCD40L cytokines alongside increased proportions of activated genital T-cells, as described in a recent study among Kenyan women (37). It needs to be acknowledged that these previous studies were of observational, cross-sectional design, conducted in different populations and measured cytokines in different sample types, while our analysis included longitudinal data from two randomized trials, which would have reduced selection bias regarding a woman’s preferred contraceptive choice. Together, our findings suggest that neither NET-EN nor DMPA-IM induce broad genital tract inflammation among women at high risk of HIV, and that these effects do not differ substantially between the two injectable contraceptives. While it is encouraging that no increased genital inflammation was observed in both groups [since genital inflammation has previously been associated with increased risk of HIV acquisition (8)], hormonal contraceptives may impact immune response differently within high-risk versus general populations (37). Since our analysis included women at high risk of HIV and STI acquisition, sexual risk behaviour was comparable between women using DMPA-IM versus NET-EN. The implications of dampened inflammation with regards to protection from other infections is likewise unknown; however, inflammation can be associated with impaired barrier function (38).

With regards to specific cytokines, rather than overall genital inflammation, we found that NET-EN initiation decreased the CCR5-binding chemokine CCL5/RANTES, as well as IL-10, both of which have been associated with increased HIV risk in SSA women (39), while DMPA-IM did not, indicating that NET-EN may have protective effects in women at high risk of HIV in SSA. Further supporting the hypothesis is the observation that DMPA-IM initiation was associated with a 1.4-fold increase of CXCL8/IL-8, a cytokine that previously has been associated with HIV risk (40), while NET-EN was not, when limiting analyses to women from the same geography. The clinical relevance of these findings is unclear, particularly since DMPA-IM use was not associated with a significantly increased HIV risk compared to the copper-IUD or LNG-implant in the ECHO Trial (9). Further investigation is needed whether subtle differences in cytokine responses translate to differences in risk of HIV acquisition, Alternatively, a randomized controlled trial with a larger sample size comparing HIV risk between DMPA-IM and NET-EN would allow higher statistical power to detect smaller differences.

In this analysis, a major difference among women using DMPA-IM versus NET-EN was the prevalence of previous pregnancies. During pregnancies with healthy birth outcomes, the relative abundance of *Lactobacillus* species (41, 42), as well as levels of most cervicovaginal cytokines, increase as gestational age progresses, thought to be due to rising endogenous progesterone throughout pregnancy (41, 43). The minimal changes seen with NET-EN use and the increase in BV-associated bacteria seen with DMPA-IM use in the current study are thus somewhat surprising; however, this could be due to the differences in synthetic progestins in injectable contraceptives and endogenous progesterone. While literature on longitudinal changes of cytokines with measurements before, during and after pregnancy is limited, DiGiulio et al. found an abrupt change in cervicovaginal microbiota post-delivery, which did not persist for longer than 1 year (42). Considering that most women in the DMPA-IM group delivered more than one year prior to study enrolment, the impact of their pregnancy on our findings is likely to be minimal.

In line with our observations regarding cervicovaginal cytokines, we found no broad differences in the vaginal microbiota composition between DMPA-IM and NET-EN users. NET-EN use was associated with decreases in the BV-associated bacterial taxa *M. hominis*, *Clostridiales*, and *Bacteroidia* spp. only, and, although not statistically significant, a higher proportion of women assigned to DMPA-IM transitioned to a more diverse microbiota composition compared to NET-EN users. Overall, DMPA-IM use appeared to be associated with a less stable microbiota, with *Lactobacillus* spp. and certain BV-associated bacteria found at a lower abundance and an increase in a higher proportion of other BV-associated bacteria, including BVAB1/*L. vaginae*, *Sneathia* spp., *P. amnii* and *Atopobium* spp., all of which are associated with increased HIV risk (44–46). Analyses integrating cytokines and bacterial relative abundances showed no distinct clustering between the two arms. Collectively, these findings support our cytokine data and suggest that both DMPA-IM and NET-EN use do not impact overall vaginal microbiota structure adversely, although NET-EN use may be superior to DMPA-IM in women at high risk of HIV acquisition due to the modest genital microbial instability seen with DMPA-IM.

The higher prevalence of *C. trachomatis* and *N. gonorrhoeae* among younger women using NET-EN and higher prevalence of HSV-2 serology among older women using DMPA-IM, albeit non-significant, is consistent with previous literature (47). On the other hand, while the prevalence of Nugent-BV in participants assigned to NET-EN was higher at baseline than in women assigned to DMPA-IM, the finding of comparable Nugent-BV prevalence after two injections with the respective contraceptives was interesting, which is in line with the increased instability in the genital microbiota seen with DMPA-IM initiation in this study. Further, the prevalence of candidiasis increased ~3-fold in both groups, suggesting that although DMPA-IM and NET-EN did not have significant adverse effects on genital inflammation and bacterial microbiota, they potentially have other non-optimal effects on sexual and reproductive health outcomes, and these effects may differ between the two progestogen-only contraceptives. These findings also support previous observations that DMPA-IM may have an immune-dampening effect (48), as women assigned to DMPA-IM did not experience an increase in inflammation despite an increase in BV prevalence.

A limitation of this study was that women included in this analyses were part of two separate cohorts, with some notable differences in population and inclusion criteria (including age and enrolment sites). While both parent trials included participants randomized to one of three contraceptive methods, participants assigned to NET-EN and DMPA-IM were not randomised against each other as part of the same trial. We however performed sub-group analyses to account for differences in age and site and found that the results are comparable to those of the complete dataset. Further, our sample size would have allowed us to only detect a 2.45-fold or larger difference in the proportion of women with genital inflammation assigned to DMPA-IM versus NET-EN, and we were thus not powered to detect any smaller, possibly not clinically relevant differences.

In conclusion, initiation of DMPA-IM and NET-EN neither increased overall genital inflammation nor substantially changed vaginal microbiota composition in Sub-Saharan African women, suggesting that NET-EN, similarly to DMPA-IM, is a viable option for contraception in African women at high risk of HIV. Further studies need to investigate whether the subtle changes we observed in cytokines and bacteria are of sufficient magnitude or duration to impact HIV risk differentially.

## Data Availability Statement

The datasets presented in this study can be found in online repositories. The names of the repository/repositories and accession number(s) can be found below: https://www.ebi.ac.uk/ena, PRJEB30774.

## Ethics Statement

The studies involving human participants were reviewed and approved by Human Research Ethics Committees at the University of Cape Town Human (HREC 371/2015 and 801/2014), University of Witwatersrand (HREC PRC 141112), KEMRI (SERU/CMR/P0014/3109), University of Washington(STUDY00000261), FHI360 (523201). Written informed consent to participate in this study was provided by the participants’ legal guardian/next of kin.

## Author Contributions

Conceived and designed the experiments for this study: HJ, A-UH, and SD. Designed the ECHO sub-study and UChoose Trial: HJ, RH, JB, J-AP, and L-GB. Enrolled the cohorts: MO, GN, TP-P, and KG. Processed samples and performed wet lab experiments: RT, BB, RB, CB, IK, CF, and SJ. Analysed the data: A-UH, SD, RT, and HJ. Wrote the manuscript: A-UH, SD, HJ. All authors contributed to the article and approved the submitted version.

## Funding

This Uchoose Trial was funded by grant NIH R01 HD083040 (PIs Jaspan and Passmore). This work and the Evidence for Contraceptive Options and HIV Outcomes (ECHO) Study were made possible by the combined generous support of the Bill & Melinda Gates Foundation (Grant OPP1032115), the American people through the United States Agency for International Development (Grant AID-OAA-A-15–00045), the Swedish International Development Cooperation Agency (Grant 2017/762965–0), the South Africa Medical Research Council, and the United Nations Population Fund. Contraceptive supplies were donated by the Government of South Africa and US Agency for International Development. Funding to support this ancillary study of biological mechanisms was from the US National Institute of Child Health and Human Development R01 HD089831 (PIs: Heffron and Jaspan). The content is the sole responsibility of the authors and does not necessarily represent the official views of the study funders. Members of the R01HD089831 ECHO Biological Mechanisms Ancillary Study Team include the Coordinating Center (University of Washington): RH, HJ (principle investigators); JB, Caitlin Scoville, Kate Heller, Harald Haugen, Colin Pappajohn.

## Conflict of Interest

The authors declare that the research was conducted in the absence of any commercial or financial relationships that could be construed as a potential conflict of interest.

## Publisher’s Note

All claims expressed in this article are solely those of the authors and do not necessarily represent those of their affiliated organizations, or those of the publisher, the editors and the reviewers. Any product that may be evaluated in this article, or claim that may be made by its manufacturer, is not guaranteed or endorsed by the publisher.

## Supplementary Material

Supplementary Table 2 Baseline cytokine levels. P-values in bold black font did not remain significant after adjusting for multiple comparisons. The p-value in red font remained significant after FDR step-down p-value adjustment.
