# Supplementary material for: Comparison of Female Genital Tract Cytokine and Microbiota Signatures Induced by Initiation of Intramuscular DMPA and NET-EN Hormonal Contraceptives - a Prospective Cohort Analysis
Source: Front Immunol. 2021 Dec 9;12:760504. doi: 10.3389/fimmu.2021.760504 (PMC8696178; doi:10.3389/fimmu.2021.760504)
Supplement: Supplementary file 1 [file DataSheet_1.pdf]

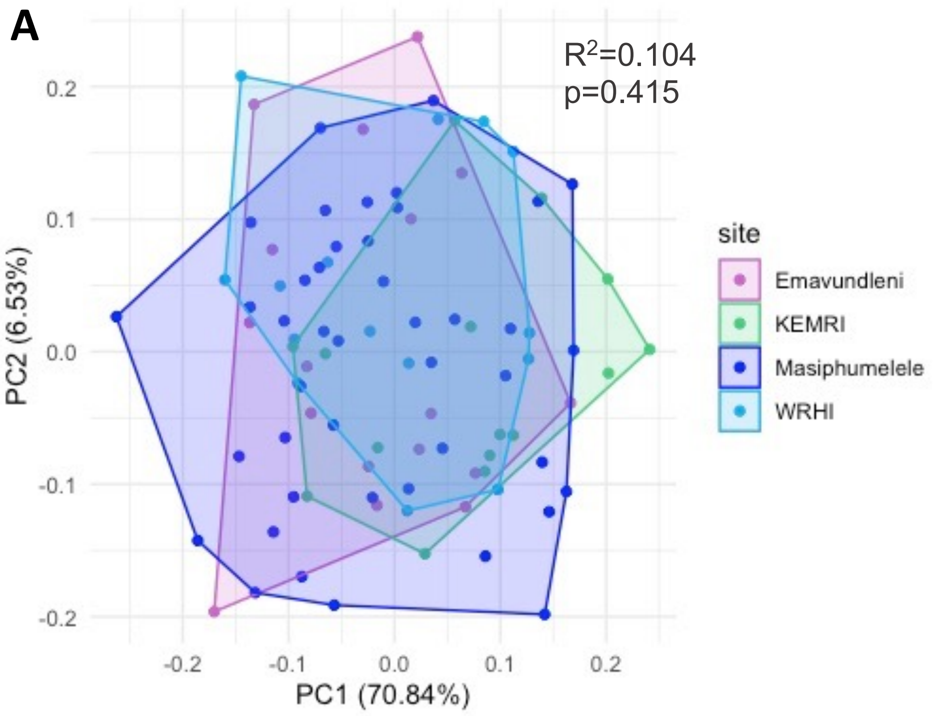

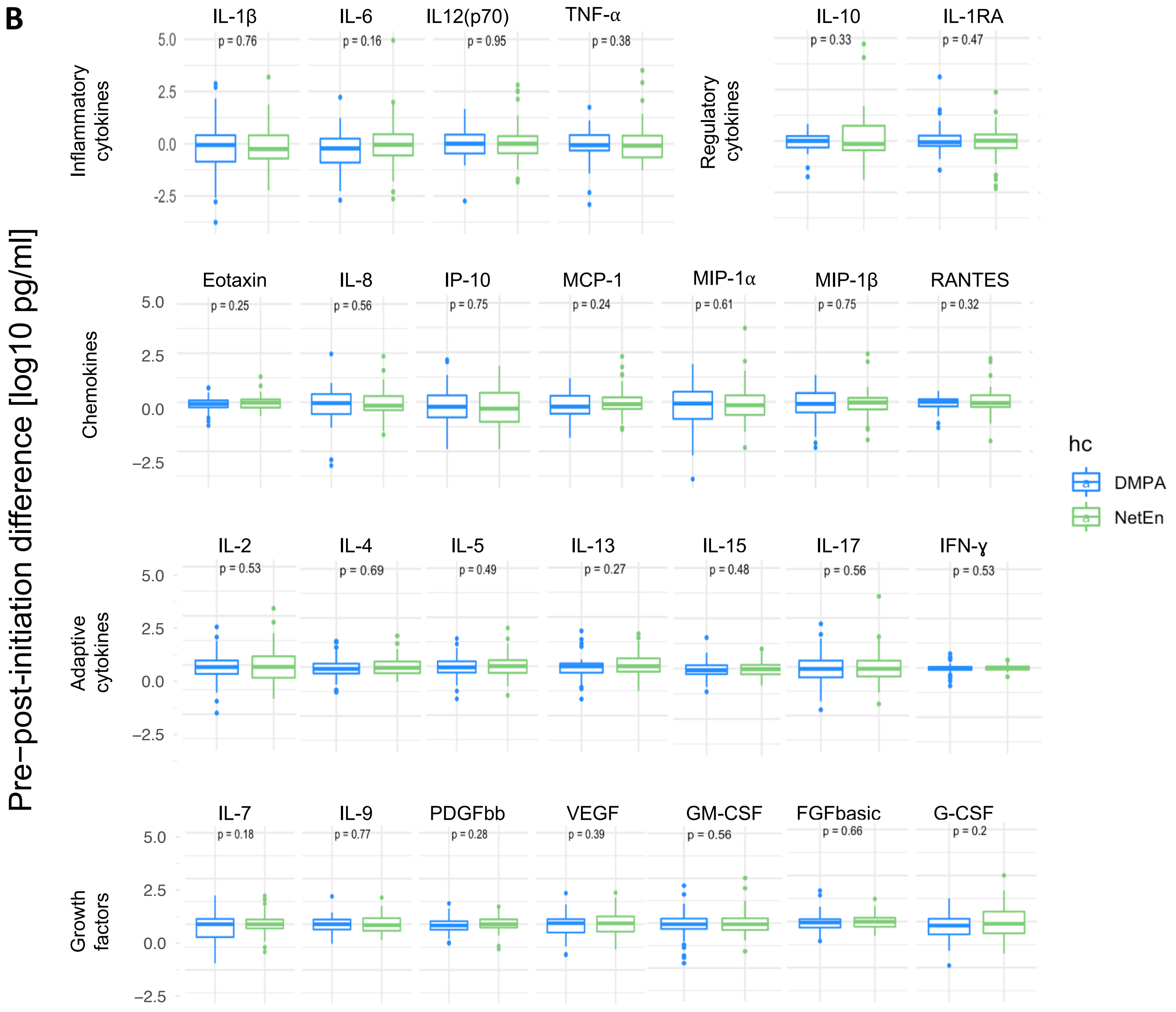

Supplementary Figure 1. Cytokines: (A) Principal component analysis plot showing no clustering by site at baseline. (B) Absolute change in log10 cytokine concentrations from baseline to Visit 2 did not differ between DMPA-IM and NET-EN users.

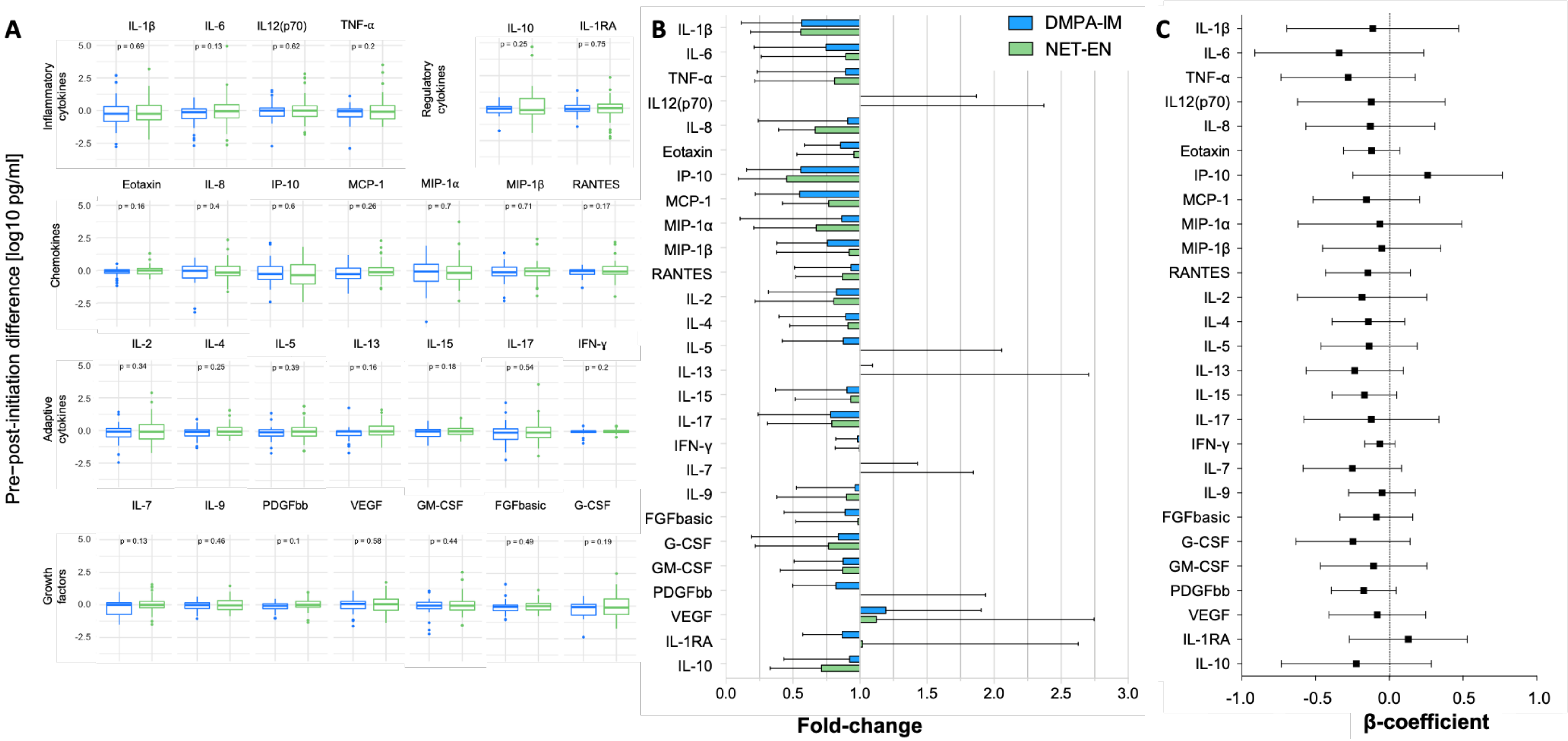

Supplementary Figure 2. Sensitivity analysis of cytokine responses in participants ≤24 years old. (A) Absolute change in log_{10} cytokine concentrations from baseline to Visit 2 in participants ≤24 years old. Participant using DMPA-IM (n=27) are shown in blue and those using NET-EN (n=43) are shown in green. (B) Fold change in log_{10} cytokine concentrations from baseline to Visit 2. (C) Multivariate linear regressions showing the association between change in log_{10} cytokine concentrations from baseline to Visit 2 in women aged ≤ 24 years using DMPA-IM as compared to NET-EN users. Associations are shown as β-coefficients and the error bars are the 95% CI.

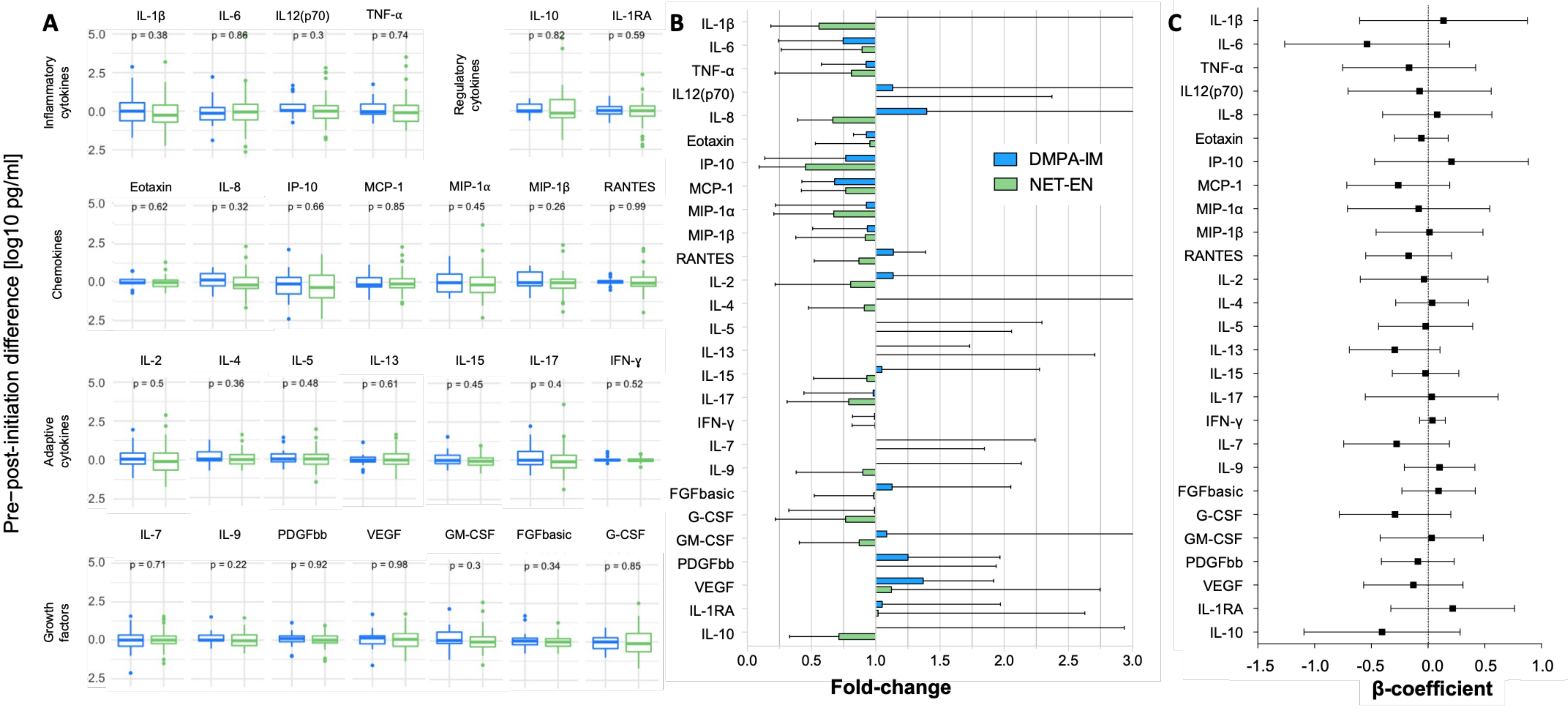

Supplementary Figure 3. Sensitivity analysis of cytokine responses in participants from Cape Town, South Africa. (A) Absolute change in log_{10} cytokine concentrations from baseline to Visit 2. Participant from Cape Town using DMPA-IM (n=17) are shown in blue and those using NET-EN (n=43) are shown in green. (B) Fold change in log_{10} cytokine concentrations from baseline to Visit 2. (C) Multivariate linear regressions showing the association change in log_{10} cytokine concentrations from baseline to Visit 2 in women from Cape Town using DMPA-IM as compared to NET-EN users. Associations are shown as β-coefficients and the error bars are the 95% CI.

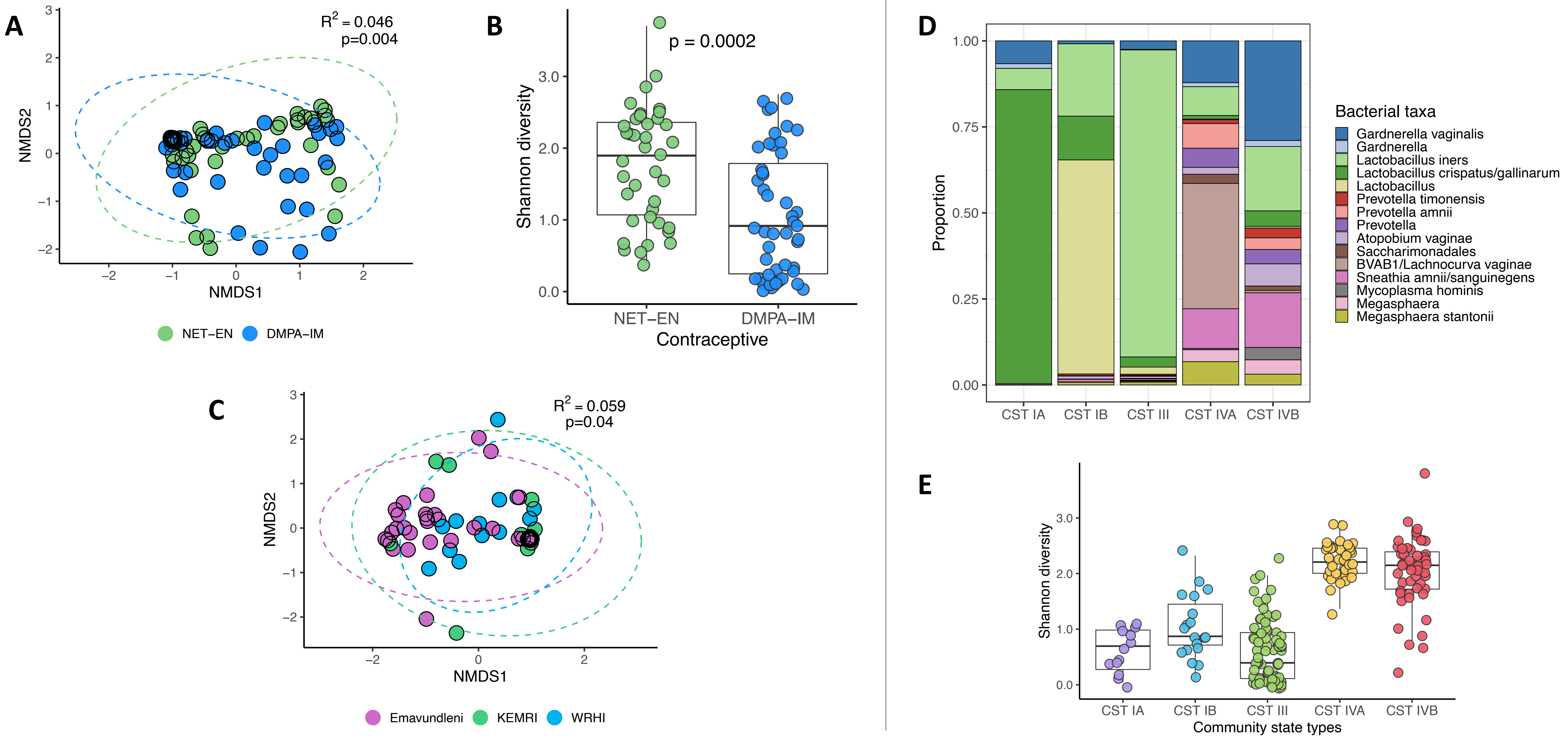

Supplementary Figure 4. Description of the genital microbiota composition. (A and C) Differences in overall genital microbiota composition (β-diversity): Principal component analysis plots showing the distinct clustering based on Bray-Curtis distances (A) between the two study arms and (C) between the different sites in the ECHO trial (B) Within-participant diversity (α-diversity) differences between participants using NET-EN or DMPA-IM. p≤0.05 were considered statistically significant. (D) Distribution of the 15 most abundant taxa among community state types (CST). CST clusters were generated by PAM clustering of Bray distances. (E) Comparison of Shannon diversity indices across CSTs. CST I: *L. crispatus*-dominant; CST III: *L. iners*-dominant; CST IVA: Diverse, BV-associated bacteria; CST IVB: Diverse, higher abundance of *L. iners* and *G. vaginalis*.

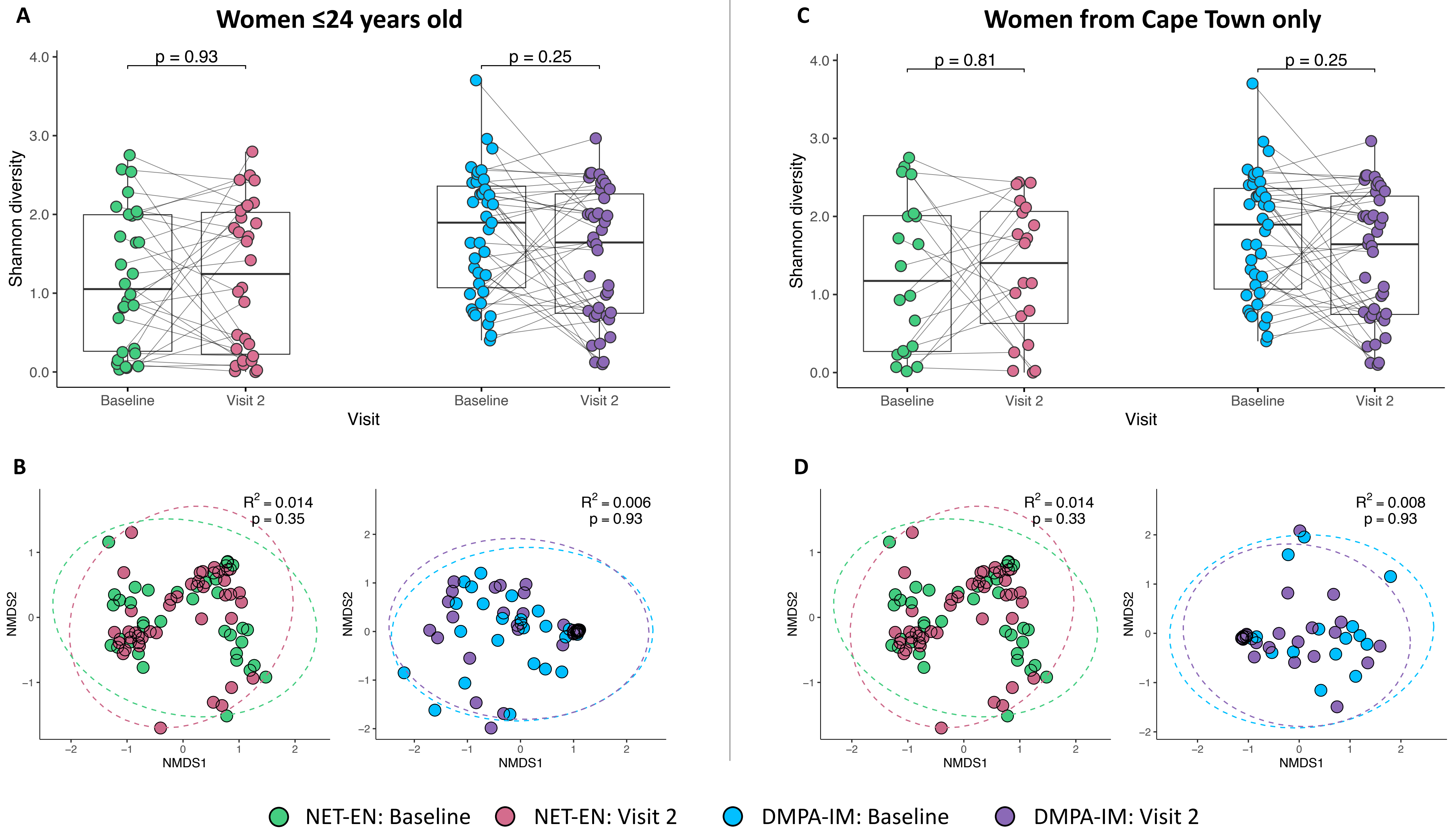

Supplementary Figure 5. Change in α-diversity and β-diversity after two injections with NET-EN and DMPA-IM use among participants ≤ 24 years old (A and B) and among participants from Cape Town, South Africa only (C and D). NMDS: Non-metric Multidimensional Scaling

Supplementary Table 3. Significant fold change differences in bacterial abundance from baseline to Visit 2 with NET-EN and DMPA-IM use.

**Bacterial taxa**	**Contraceptive**	**log2FoldChange**	**Standard error**	**p value***
Mycoplasma hominis	NET-EN	-2.657	0.577	<0.001
Clostridiales	NET-EN	-1.757	0.416	<0.001
Bacteroidia	NET-EN	-1.523	0.394	<0.001
Ureaplasma urealyticum	DMPA-IM	-1.567	0.492	<0.001
Peptoniphilus asaccharolyticus/grossensis/harei	DMPA-IM	-1.288	0.475	0.001
Staphylococcus	DMPA-IM	-1.251	0.555	0.002
Ezakiella	DMPA-IM	-1.250	0.511	<0.001
Peptostreptococcus anaerobius	DMPA-IM	-0.897	0.446	0.001
Prevotella bivia/denticola	DMPA-IM	-0.837	0.502	<0.001
Lactobacillus	DMPA-IM	-0.313	0.747	0.001
Sneathia	DMPA-IM	0.664	0.441	<0.001
Atopobium	DMPA-IM	0.797	0.455	<0.001
Fastidiosipila	DMPA-IM	1.191	0.450	<0.001
Lachnovaginosum BVAB1	DMPA-IM	1.453	0.587	<0.001
Prevotella amnii	DMPA-IM	1.468	0.510	<0.001
Sneathia sanguinegens	DMPA-IM	1.809	0.527	<0.001
Sneathia amnii	DMPA-IM	3.046	0.564	0.001

* p values based on a Wald test using parameters estimated by maximum likelihood, and adjusted for multiple comparisons using the FDR/Benjamini-Hochberg method
